# Seasonal variations of urate in a Swedish adult population

**DOI:** 10.1007/s10067-017-3591-z

**Published:** 2017-03-10

**Authors:** Axel Åkerblom, Johanna Helmersson-Karlqvist, Tomas Weitoft, Anders Larsson

**Affiliations:** 1 Department of Medical Sciences, Cardiology, Uppsala University, Uppsala, Sweden; 2 Uppsala Clinical Research Center, Uppsala, Sweden; 3 Department of Medical Sciences, Clinical Chemistry, Uppsala University, Uppsala, Sweden; 4 Section of Rheumatology, Center of Research and Development, Uppsala University/Region of Gävleborg, Gävle, Sweden

**Keywords:** Biological markers, Circannual variation, Gout, Humans, Urate, Uric acid

## Abstract

Seasonality in the incidence and prevalence of gout has previously been reported but the cause of this seasonality in gout is not explained. The aim of this study was to evaluate possible seasonal variations of urate in a large unselected Swedish adult population. We analyzed 170,915 urate test results from patients at a tertiary care hospital between 2000 and 2016. The results were divided according to sex and sampling month of the year. The median urate values were overall higher in males compared to females and both males and females had peak urate concentrations in the summer months (June–August). There is a seasonal pattern for urate concentrations in a large Swedish population similar to the previously reported seasonality for gout. This may be clinically important and could contribute to the circannual variation of gout. The seasonal pattern should be recognized when evaluating patient results both in clinical practice and in research studies.

## Introduction

Gout is one of the most commonly diagnosed rheumatic diseases and it is estimated that the prevalence is 1–2% in an adult population [1, 2]. Elderly individuals and males are more affected [2]. It is often recommended that the urate lowering treatment of gout patients should be monitored by urate concentrations. The treatment goals are usually to reduce serum urate <360 μmol/L (6 mg/dL) if the patient do not have tophi and <300 μmol/L (5 mg/dL) in patients with tophi [3–5]. Gout is often divided into primary gout and secondary gout. Secondary gout is more common and is mainly caused by life style factors, pharmaceutical treatments or other diseases [6, 7]. The prevalence of gout has gradually increased over the last decades [8]. This has been attributed to among other factors the increased life expectancy but also to the worldwide increase in obesity and increased body mass index (BMI) [9]. Alcohol intake may also increase urate production and reduce the elimination while diuretics such as thiazides and acetylic acid may reduce the elimination of urate [10, 11].

Urate or uric acid is a metabolite from the purine degradation and is ubiquitous in humans. It is the major determinant of the urate concentration [12]. Humans have a limited capacity to degrade urate and the elimination is mainly through the kidneys or the gastrointestinal tract [13]. Increased levels of urate can consequently either be due to increased synthesis or decreased elimination of urate. When blood and tissues become oversaturated with uric acid, crystallization will occur [14, 15]. The crystallization occur in tissues and may cause flares of inflammation in predominantly joints, bursas, tendon sheaths and surrounding soft tissues. Although, hyperuricemia is the single most important risk factor for gout, only a minority of patients with hyperuricemia develop gout. The association between urate concentrations and gout is thus complex. Nevertheless, measuring the urate concentration is crucial for diagnosing and monitoring of patients with gout and it is thus important to be aware of natural fluctuations of urate in the population.

The aim of this study was to identify possible seasonal variations in plasma urate and, if so, describe the seasonal pattern. We also aimed to correlate the seasonal variation of urate with the previously reported seasonality of gout with higher incidence during the summer period in countries located in the northern hemisphere [16, 17].

## Methods

### Samples

All urate samples between May 15th, 2000, and December 31st, 2015, analyzed at the central lab in the Uppsala University Hospital, Uppsala, Sweden, *in Uppsala County, Sweden,* were recorded in the laboratory information system (FlexLab). All tests were performed on fresh blood samples and only test reports with a valid quantitative result and information on age and sex were included in further analyses. In total, 170,915 test results were included. The study was approved by the local ethical board (01–367) and complied with the World Medical Association Declaration of Helsinki regarding ethical conduct of research.

### Statistical analysis

The difference between males and females was tested with Mann-Whitney U test. Differences between calendar months were tested with Kruskal-Wallis ANOVA. The results were reported for men and women separately. A *p* value of <0.05 was considered as significant.

## Results

### Urate values in males and females

A total of 170,915 urate test results (80,050 samples from males and 90,865 from females) were collected. The median urate concentration for the males was 377 μmol/L (Interquartile range (IQR) 303–462 μmol/L) and for the females 294 μmol/L (IQR 232–374 μmol/L). There were significantly higher median concentrations in males compared to women (*p* < 0.00001).

### Seasonal variation of urate

**Fig. 1:**
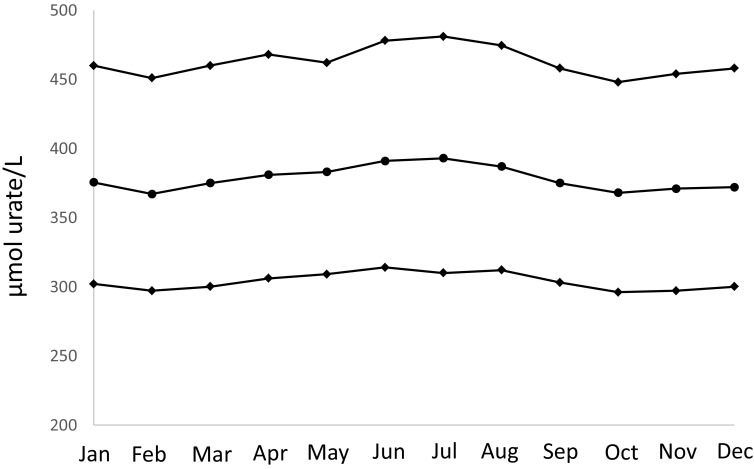
Median and interquartile ranges for urate values in μmol/L for males divided according to month (*P* < 0.0001)

**Fig. 2:**
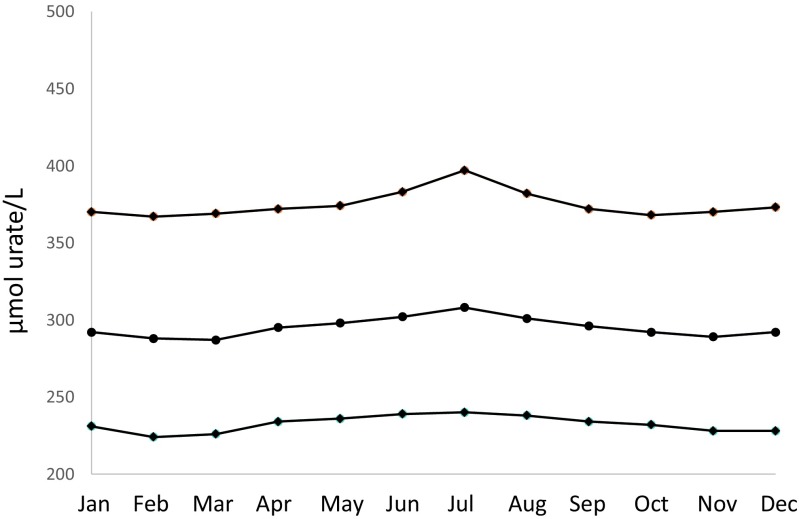
Median and interquartile urate values in μmol/L for females divided according to month (*P* < 0.0001)

Urate concentrations were significantly different between the calendar months in both men (*P* < 0.0001) and women (*P* < 0.0001) with the highest concentrations during the summer months (Figs. 1 and 2 and Table 1). The urate levels in June–August were significantly higher in both men and women when each month was compared.

**Table 1:** Urate values for individual months for females and males, respectively. The values are presented as min, first quartile, median, third quartile, and max values

	Min	First quartile	Median	Third quartile	Max
Females					
January	15	231	292	370	1303
February	6	224	288	367	1580
March	10	226	287	369	1587
April	6	234	295	372	1356
May	8	236	298	374	1164
June	9	239	302	383	1554
July	6	240	308	397	1423
August	10	238	301	382	1244
September	6	234	296	372	1535
October	7	232	292	368	1634
November	26	228	289	370	1305
December	8	228	292	373	1317
Males					
January	5	302	376	460	1467
February	5	297	367	451	1172
March	6	300	375	460	1130
April	11	306	381	468	1252
May	8	309	383	462	1404
June	5	314	391	478	1334
July	6	310	393	481	1313
August	7	312	387	475	1420
September	6	303	375	458	1738
October	14	296	368	448	1856
November	5	297	371	454	1385
December	5	300	372	458	1410

## Discussion

Gout is the most common inflammatory arthritis associated with crystal formation. The deposition of urate crystals is a consequence of supersaturated levels of soluble urate in combination with local factors that may modify the crystal formation. The point of saturation of urate in plasma is considered to be approximately 6.8 mg/dl (405 μmol/L) [15]. The aim of urate lowering therapies is to reduce the urate level below the point of saturation in order to prevent gout flares and symptoms. Most recommendations aim to reduce urate <360 μmol/L (6 mg/dL) to hinder the formation of new urate crystals [18]. In patients with tophi a limit of <300 μmol/L (5 mg/dL) [19]. The present study shows a clinically important seasonal variation for plasma urate. The median urate levels in July are approximately 20 μmol/L higher than the median urate levels in February and the upper quartile approximately 30 μmol/L higher with similar patterns for both gender. In our opinion the seasonal variation should be taken into account when reviewing urate test results and a lower treatment threshold should be used in February in comparison with in July. Additionally, if a patient has urate concentration of 350 μmol/L in the beginning of the year, it is likely that the urate levels may exceed 360 μmol/L during the summer months. Consequently, a more intensive urate lowering therapy may be applied during the summer as well.

The increased levels of urate seen during the summer months and the smaller peak observed around December–January may be due to variation in the diet. The traditional Christmas-New year celebrations in Sweden are usually associated with increased consumption of food rich in meat, alcohol, and beer. Studies have shown that the holiday season is a critical period for weight gain as an effect of the increased food intake [20]. High meat consumption and alcohol intake especially beer is known to be risk factors for gout. The warm weather during the summer months is associated with increased intake of sweetened beverages and fruits. Studies have shown that fructose intake from sugar-sweetened beverages causes transient increases in plasma urate [21]. Other dietary factors are seafood, coffee, and dairy product consumption. Even if Sweden in general has a high coffee consumption, we do not know of any seasonal variation that could be related to increased urate levels during the summer months.

The seasonal urate variation with high levels during the summer months is in agreement with two previous studies on gout incidence from the northern hemisphere [16, 17] but contradictory to a US study [22]. The UK and Korea studies show higher gout incidence during the same time of the year. Considering that hyperuricemia is the strongest risk factor for gout, it seems reasonable that the higher urate levels during the summer months may contribute to the higher incidence of gout. This would also implicate that a reduction of urate levels during the summer months would reduce the increased incidence of gout during the same time period. For patients using urate lowering therapies, such as allopurinol, higher doses might be needed during the summer time. Although the increased levels of urate were consistently observed during the summer, the current study was not designed to determine the cause to this finding. Neither was the study designed to firmly associate the increased levels with the incidence of gout. The clinical reason for obtaining the urate samples was not available. Although the urate samples were likely obtained in similar clinical situations, we cannot rule out that the circannual higher incidence of gout during summer may have affected the causes for heightened urate concentration. Nonetheless, the amount of samples greatly exceeds the clinical manifestations of gout in the current population, and hence, median plasma urate is hardly changed by increased gout incidence and, consequently, urate are likely obtained in far more clinical situations than only to diagnose possible gout. Reduced glomerular filtration rate leads to increased urate levels. Further studies are warranted that focus on the seasonal variation of urate in patients with reduced GFR levels.

In conclusion, urate shows a clear seasonal variation with higher levels during the summer months which coincides with the seasonal variation previously reported for gout. This seasonal urate variation should be taken into account when monitoring gout patients.
